# Long‐Term Effects of Myocardial Injury on 1‐Year Outcomes in Hospital Survivors With COVID‐19: A Prospective Cohort Study

**DOI:** 10.1002/mco2.71119

**Published:** 2026-11-03

**Authors:** Wu He, Qiongyao He, Ke Xu, Gen Li, Yongcui Yan, Bo Yu, Yujing Su, Junfang Wu, Hong Wang, Dao Wen Wang

**Affiliations:** ^1^ Division of Cardiology, Department of Internal Medicine, Hubei Key Laboratory of Genetics and Molecular Mechanisms of Cardiological Disorders, Tongji Hospital, Tongji Medical College Huazhong University of Science and Technology Wuhan China; ^2^ Department of Endocrinology, Department of Internal Medicine, Tongji Hospital, Tongji Medical College Huazhong University of Science and Technology Wuhan China

**Keywords:** ACEI/ARB, all‐cause mortality, COVID‐19, health‐related quality of life, major adverse cardiovascular events, myocardial injury

## Abstract

Myocardial injury is both a common complication among hospitalized coronavirus disease 2019 (COVID‐19) patients and a major predictor of adverse clinical outcomes. However, its long‐term effects remain poorly defined. In this prospective cohort study of 2185 COVID‐19 survivors (531 with myocardial injury), we assessed 1‐year post‐discharge outcomes using Kaplan–Meier analysis and multivariable Cox regression before and after propensity score matching. Myocardial injury was associated with higher all‐cause mortality (adjusted hazard ratio [aHR] 3.14) and major adverse cardiovascular events (MACE) (aHR 5.22). Long‐term use of angiotensin‐converting enzyme inhibitors/angiotensin II receptor blockers (ACEI/ARB) was associated with reduced mortality (aHR 0.44) and MACE (aHR 0.58) in the myocardial injury group. At 1 year, 71.5% of survivors reported persistent symptoms—most commonly fatigue and sleep difficulties—and those with myocardial injury had poorer quality of life. This study provides the prospective evidence from China's Omicron era on the prognostic value of myocardial injury, combining hard clinical endpoints with patient‐reported outcomes and the first analysis of the ACEI/ARB association in this population. These findings highlight the prognostic importance of myocardial injury and the potential role of ACEI/ARB therapy in post‐discharge management, supporting targeted follow‐up strategies for this high‐risk population.

## Graphical abstract

Among COVID‐19 patients, myocardial injury was associated with higher 1‐year mortality and MACE, whereas long‐term ACEI/ARB therapy was associated with lower risks. At 1 year, 71.5% of survivors reported symptoms; myocardial injury was linked to more sequelae and poorer quality of life.

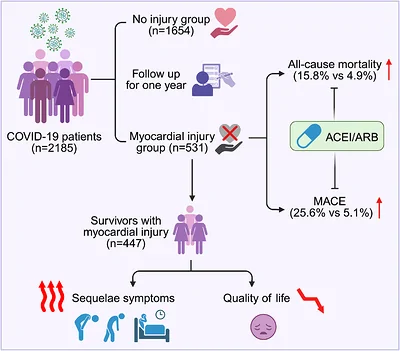

## 1 Introduction

The global coronavirus disease 2019 (COVID‐19) pandemic has profoundly affected global health and resulted in significant morbidity and mortality, affecting hundreds of millions of people worldwide [1]. Among the myriad complications associated with COVID‐19, cardiovascular manifestations, particularly myocardial injury, have emerged as critical determinants of patient outcomes and become a significant concern, frequently observed in patients hospitalized with COVID‐19 [2, 3].

Clinically, myocardial injury in COVID‐19 is often identified by elevated levels of cardiac biomarkers, such as high‐sensitivity cardiac troponin I (hs‐cTnI), and is associated with a spectrum of cardiovascular manifestations, ranging from subtle myocardial inflammation to acute coronary syndromes and fulminant myocarditis [4, 5]. These manifestations can exacerbate pre‐existing cardiovascular conditions or trigger new cardiac events [6]. Myocardial injury during COVID‐19 is associated with increased in‐hospital mortality and adverse cardiovascular events, positioning it as a crucial factor in patient prognosis [7, 8]. Additionally, some patients exhibit persistent cardiovascular symptoms several months after the initial severe acute respiratory syndrome coronavirus 2 (SARS‐CoV‐2) infection, a condition often characterized as long COVID [9, 10].

Despite the growing body of evidence on the acute consequences of myocardial injury in COVID‐19 patients [11, 12, 13], the long‐term post‐discharge trajectory remains incompletely characterized, particularly in the Omicron era in China, where combined clinical and patient‐reported outcomes and the specific angiotensin‐converting enzyme inhibitors/angiotensin II receptor blockers (ACEI/ARB) association have not been systematically examined [14]. Understanding the extended impact of myocardial injury on mortality and major adverse cardiovascular events (MACE) is essential for informing clinical follow‐up and management strategies. Furthermore, the persistent symptom burden and quality of life among survivors, particularly those with a history of myocardial injury, require thorough investigation to address ongoing healthcare needs [15].

Our study aims to address this knowledge gap by examining the 1‐year outcomes of patients with and without myocardial injury who were hospitalized for COVID‐19. Specifically, we assessed all‐cause mortality and MACE in the COVID‐19 cohort within 1 year of discharge through follow‐up. In addition, we assessed the persistent symptoms and quality of life of survivors 1 year after discharge through a structured questionnaire. This study provides insight into the prognostic impact of myocardial injury sustained during acute COVID‐19 infection.

## 2 Results

### 2.1 Baseline Characteristics

The study included patients who were admitted between December 2022 and February 2023 and discharged through March 2023, totaling 3471 individuals who underwent hs‐cTnI testing. From this initial cohort, 781 patients under 18 years of age and 312 patients who died during hospitalization were excluded. Consequently, 2378 laboratory‐confirmed COVID‐19 patients who were discharged from the hospital were included in the study. All discharged patients met the standardized discharge criteria outlined in the “Chinese clinical guidance for COVID‐19 pneumonia diagnosis and treatment” issued by the National Health Commission of China. During the follow‐up of the 2378 discharged survivors, 66 patients declined to participate, and 127 patients were lost to follow‐up within the 1‐year period. Ultimately, 2185 COVID‐19 patients were included in the final analysis (Figure 1). Among the 2378 discharged survivors, 66 (2.8%) declined participation and 127 (5.3%) were lost to follow‐up, yielding a final follow‐up completion rate of 91.9%.

**FIGURE 1:**
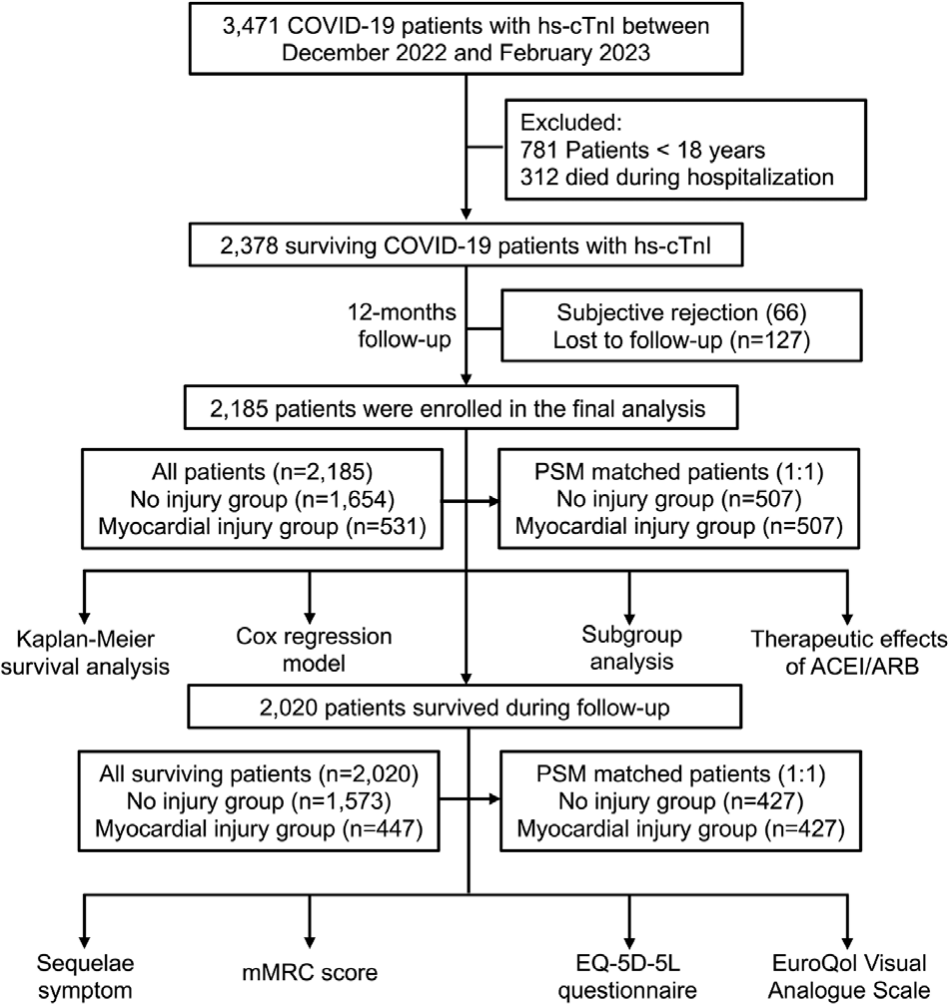
The flowchart of study design.

In contrast to our previous analysis of the acute in‐hospital cohort (*n* = 2690) [11], which included patients who died during hospitalization, the current prospective study focuses on discharged survivors (*n* = 2185), enabling assessment of post‐discharge outcomes and longitudinal symptom burden. Among all patients, 531 (24.3%) were classified into the myocardial injury group with hs‐cTnI levels exceeding the upper reference limit, while 1654 (75.7%) were in the no injury group. Initial comparisons between these groups revealed significant differences in several baseline characteristics. Patients with the myocardial injury were, on average, older and exhibited a higher prevalence of chronic heart failure. Additionally, there were notable differences in some laboratory parameters, including platelet count, alanine aminotransferase (ALT), and blood urea nitrogen (BUN) levels, with all these differences reaching statistical significance (*p* < 0.05). The median hs‐cTnI levels were 46.4 pg/mL (interquartile range [IQR] 32.7–110.0) for the myocardial injury group and 4.6 pg/mL (IQR 2.0–9.0) for the no injury group (*p* < 0.05). To address potential confounding factors and ensure a more balanced comparison between the two groups, a 1:1 propensity score matching (PSM) was conducted. This statistical technique matched patients from the myocardial injury group with those from the no injury group based on key baseline characteristics, creating a matched cohort of 507 patients in each group. Following PSM, the two groups were well‐balanced with no significant differences observed in the baseline characteristics (all *p* > 0.05), except for hs‐cTnI levels, which remained higher in the myocardial injury group, as expected due to its defining nature. The baseline characteristics of the groups before and after PSM are shown in Table 1 and Table S1.

**TABLE 1:** Baseline of COVID‐19 patients in myocardial injury group and no injury group.

		Unmatched			PSM (1:1)		
	All patients (*n* = 2185)	No injury group (*n* = 1654)	Myocardial injury group (*n* = 531)	*p*	No injury group (*n* = 507)	Myocardial injury group (*n* = 507)	*p*
Gender: Male, *n* (%)	1281 (58.6)	946 (57.2)	335 (63.1)	0.016	317 (62.5)	323 (63.7)	0.696
Age, years	67 (56–76)	66 (55–75)	72 (60‐81)	< 0.001	71 (59‐80)	71 (59‐80)	0.967
Age ≥ 70 years, *n* (%)	965 (44.2)	671 (40.6)	294 (55.4)	< 0.001	271 (53.5)	271 (53.5)	> 0.999
Age < 70 years, *n* (%)	1220 (55.8)	983 (59.4)	237 (44.6)	< 0.001	236 (46.5)	236 (46.5)	> 0.999
Smoking, *n* (%)	375 (17.2)	281 (17.0)	94 (17.7)	0.704	87 (17.2)	90 (17.8)	0.804
Drinking, *n* (%)	231 (10.6)	176 (10.6)	55 (10.4)	0.854	49 (9.7)	52 (10.3)	0.753
Hypertension (%)	761 (34.8)	572 (34.6)	189 (35.6)	0.671	169 (33.3)	184 (36.3)	0.323
CHD (%)	286 (13.1)	201 (12.2)	85 (16.0)	0.022	76 (15.0)	71 (14.0)	0.656
CHF (%)	159 (7.3)	102 (6.2)	57 (10.7)	< 0.001	46 (9.1)	44 (8.7)	0.825
T2DM (%)	433 (20.3)	341 (20.6)	102 (19.2)	0.702	112 (22.1)	96 (18.9)	0.213
CKD (%)	289 (13.2)	198 (12.0)	91 (17.1)	0.002	94 (18.5)	85 (16.8)	0.459
Solid tumors, *n* (%)	176 (8.1)	133 (8.0)	43 (8.1)	0.967	37 (7.3)	41 (8.1)	0.637
Temperature, °C	36.5 (36.4–36.7)	36.5 (36.4–36.8)	36.5 (36.4–36.7)	0.660	36.5 (36.4–36.8)	36.5 (36.4–36.7)	0.443
RR, breaths/min	20 (20–20)	20 (20–20)	20 (20–20)	0.483	20 (20–20)	20 (20–20)	0.241
Pulse, beats/min	83 (78–92)	84 (78–92)	82 (78–90)	0.214	83 (78–92)	82 (78–90)	0.516
SBP, mmHg	126 (118–137)	126 (117–137)	126 (120–137)	0.355	127 (118–138)	126 (119–138)	0.796
DBP, mmHg	78 (71–84)	78 (71–84)	78 (72–85)	0.822	78 (70–84)	78 (72–85)	0.986
hs‐cTnI, pg/mL	7.1 (2.6–23.9)	4.6 (2.0–9.0)	46.4 (32.7–110.0)	< 0.001	2.3 (1.9–5.4)	46.1 (32.5–107.8)	< 0.001

*Note*: Data are presented as median (IQR) or *n* (%).

Abbreviations: CHD, coronary heart disease; CHF, chronic heart failure; CKD, chronic kidney disease; hs‐cTnI, high‐sensitivity cardiac troponin I; PSM, propensity score matching; RR, respiratory rate; SBP, systolic blood pressure; T2DM, type 2 diabetes.

### 2.2 Long‐Term Medication Use

We conducted a follow‐up on long‐term medication uses among all COVID‐19 patients after discharge, focusing on key cardiovascular and metabolic treatments. The medications included angiotensin‐converting enzyme inhibitors or angiotensin II receptor blockers (ACEI/ARB), diuretics, calcium channel blockers (CCB), β‐blockers, statins, metformin, nitrates, anticoagulants, and antiplatelet agents. Our analysis revealed no significant differences in the patterns of long‐term medication use between the myocardial injury and no injury groups, both before and after PSM (all *p* > 0.05). This consistency in medication usage suggests that post‐discharge pharmacological management was similar for both groups, regardless of the presence of myocardial injury during hospitalization. Table S2 provides a detailed comparison of medication use between the groups.

### 2.3 Outcomes During Follow‐Up

We compared the 1‐year all‐cause mortality and MACE between the myocardial injury and no injury groups. In the myocardial injury group, 84 patients (15.8%) died from any cause, and 136 patients (25.6%) experienced MACE. In contrast, the no injury group reported 81 deaths (4.9%) and 85 MACE occurrences (5.1%). The Kaplan–Meier survival curve and proportional Cox regression analyses (all satisfied the proportional hazards assumption, with Schoenfeld residual global *p* > 0.05 for all models) indicated a significantly higher risk in the myocardial injury group for all‐cause mortality (log‐rank *p* < 0.001; adjusted hazard ratio [aHR], 3.14; 95% confidence interval [CI], 2.31–4.27; Figure 2A) and MACE (log‐rank *p* < 0.001; aHR, 5.22; 95% CI, 3.97–6.86; Figure 2B) compared to the no injury group. Notably, these findings were consistent even after PSM, with the myocardial injury group continuing to show higher rates of all‐cause mortality (log‐rank *p* < 0.001; aHR, 2.79; 95% CI, 1.82–4.28; Figure 2C) and MACE (log‐rank *p* < 0.001; aHR, 4.92; 95% CI, 3.28–7.38; Figure 2D). Further analysis of the components of MACE, including cardiovascular death, nonfatal myocardial infarction, non‐fatal stroke, and hospitalization for heart failure, revealed consistently higher incidence rates in the myocardial injury group compared to the no injury group, both before and after PSM (Figure S1A–H).

**FIGURE 2:**
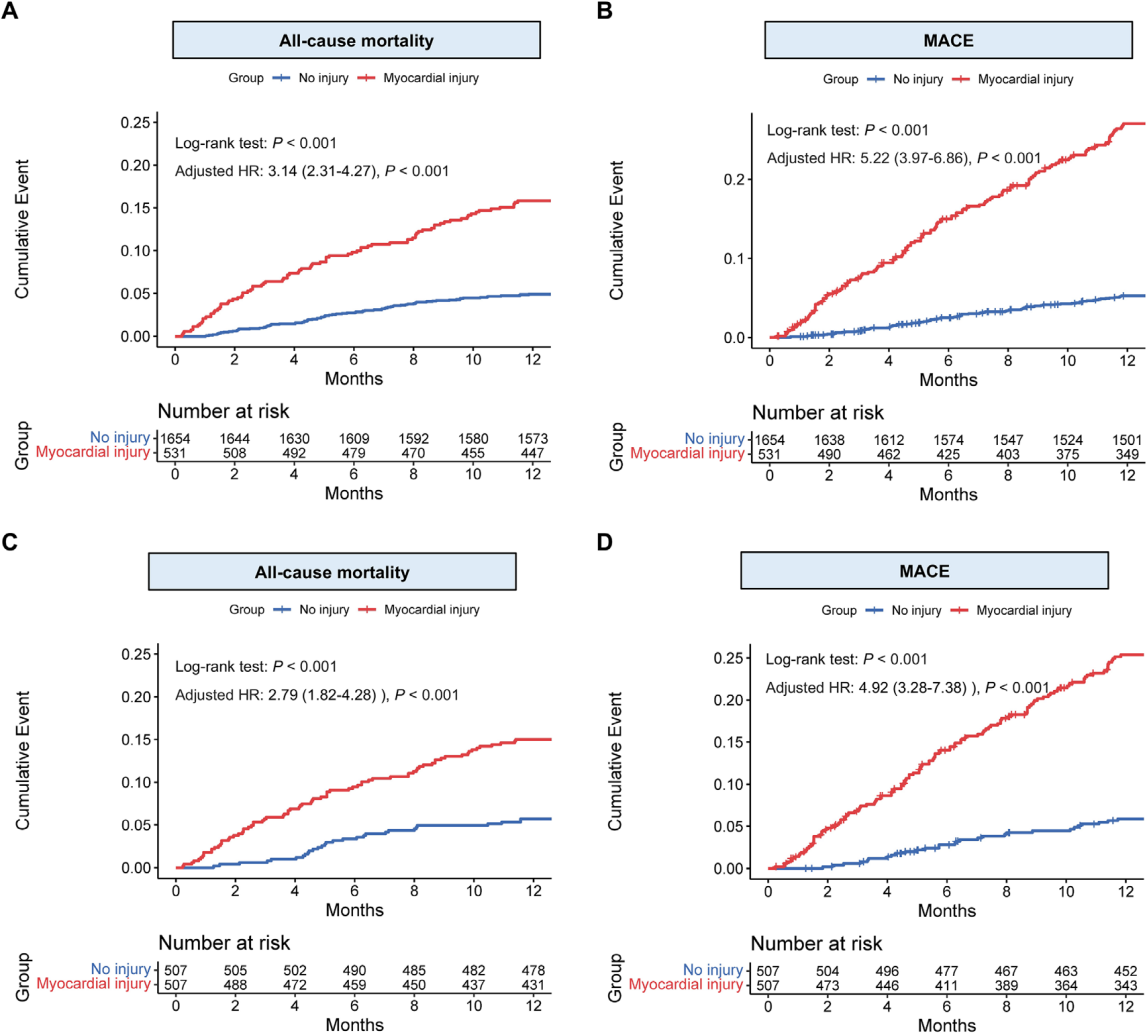
Survival analysis of 1‐year clinical outcomes for COVID‐19 patients in myocardial injury and no injury groups before and after PSM. (A) The Kaplan–Meier survival curve of all‐cause mortality for COVID‐19 patients in myocardial injury group and no injury group before PSM. (B) The Kaplan–Meier survival curve of major adverse cardiovascular events (MACE) for COVID‐19 patients in myocardial injury group and no injury group before PSM. (C) The Kaplan–Meier survival curve of all‐cause mortality for COVID‐19 patients in myocardial injury group and no injury group after PSM. (D) The Kaplan–Meier survival curve of MACE for COVID‐19 patients in myocardial injury group and no injury group after PSM. The decline in the number at risk reflects administrative censoring as patients completed their 12‐month follow‐up; no patients were censored before their actual follow‐up completion date.

The subgroup analyses of all‐cause mortality and MACE were based on stratified factors and the selection of prespecified subgroups, including age (< 70 or ≥ 70 years), gender, smoking status, hypertension, coronary artery disease, chronic heart failure, type 2 diabetes, ALT levels (< 40 or ≥ 40 U/L), estimated glomerular filtration rate (eGFR, < 90 or ≥ 90 mL/min/1.73m^2^), and D‐dimer levels (< 1 or ≥ 1 µg/mL). The analyses demonstrated consistent effects across these subgroups (Figure 3A,B). For all‐cause mortality, significant subgroup interactions were observed for smoking and CHF, with myocardial injury posing a particularly severe risk factor in smokers (aHR, 5.19; 95% CI, 2.56–10.51; *p* < 0.001) and patients with CHF (aHR, 5.55; 95% CI, 2.00–15.41; *p* < 0.001). The risk of MACE was significantly higher in patients younger than 70 years (aHR, 8.95; 95% CI, 5.98–13.38; *p* < 0.001) and those with ALT levels ≥ 40 U/L (aHR, 7.37; 95% CI, 3.95–13.75; *p* < 0.001).

**FIGURE 3:**
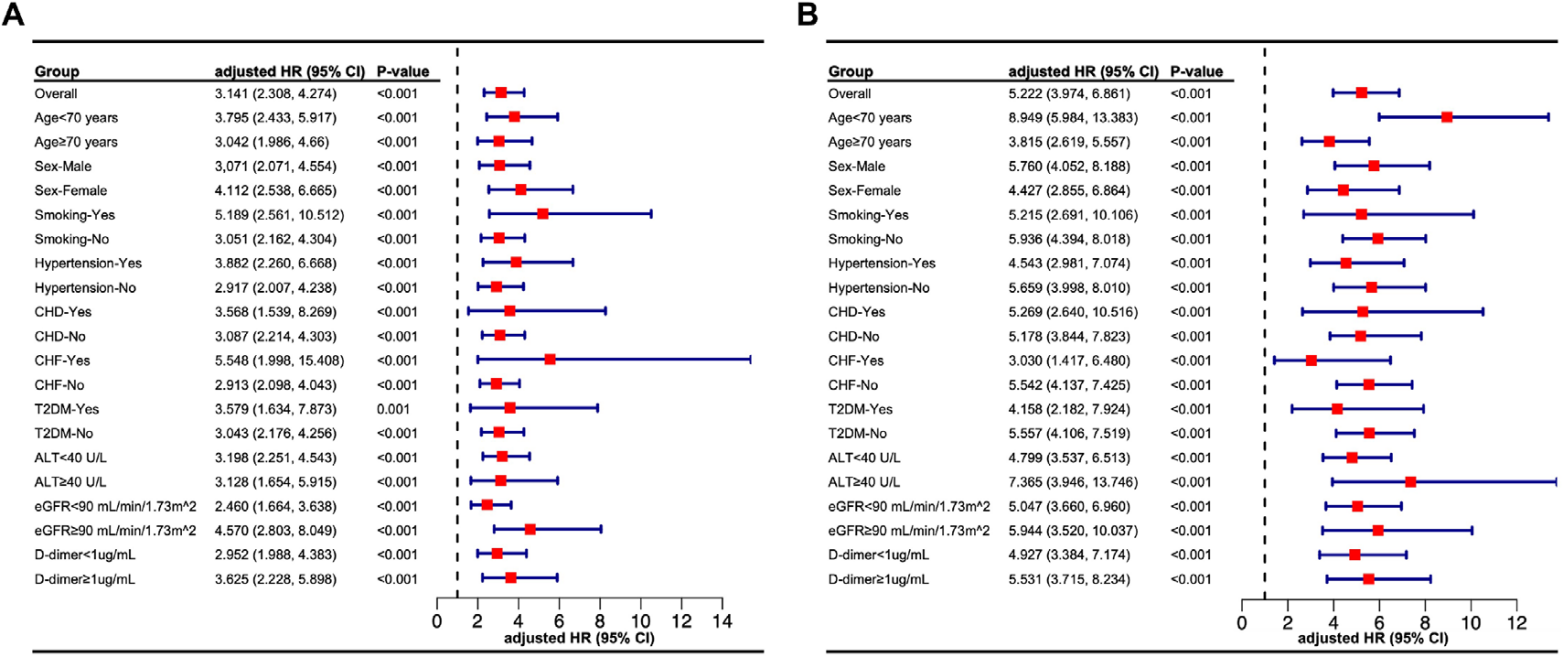
One‐year clinical outcomes in select prespecified subgroups. (A) All‐cause mortality in subgroups. (B) Major adverse cardiovascular events (MACE) in subgroups.

To further identify the prognostic performance of absolute hs‐cTnI levels on clinical outcomes in COVID‐19 patients, we stratified all patients, in both no injury and myocardial injury groups, into quartiles based on hs‐cTnI levels (both before and after PSM). The Kaplan–Meier curves illustrated that COVID‐19 patients in the highest hs‐cTnI quartile had the highest cumulative incidence of all‐cause mortality and MACE (all *p* < 0.05; Figure S2A–F). Consistent patterns were observed using restricted cubic splines (RCS) functions within the entire COVID‐19 cohort, demonstrating a pattern similar to that of quartile analysis (*p* for overall < 0.001 for both all‐cause mortality and MACE; Figure 4A,B).

**FIGURE 4:**
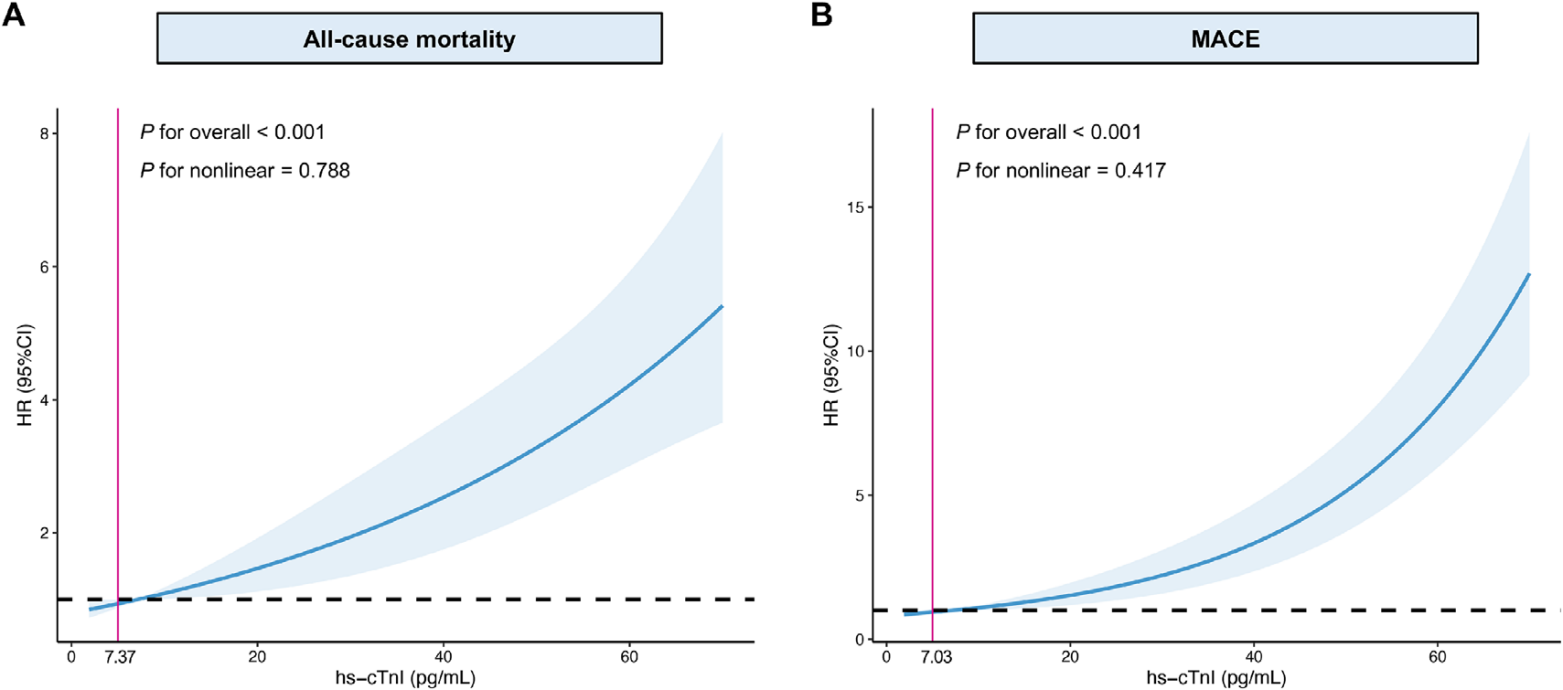
Risk of 1‐year clinical outcomes in COVID‐19 patients according to hs‐cTnI levels using restricted cubic splines. (A) All‐cause mortality. (B) Major adverse cardiovascular events (MACE).

### 2.4 Impact of ACEI/ARB Treatment in Patients With Myocardial Injury

Our and other previous observations have demonstrated that ACEI/ARB significantly reduce in‐hospital mortality in patients with COVID‐19 complicated by myocardial injury [11, 16, 17]. Building on these findings, we sought to assess the impact of long‐term ACEI/ARB use on 1‐year clinical outcomes in COVID‐19 survivors, both with and without myocardial injury. The Kaplan–Meier survival analyses were conducted to evaluate the effect of long‐term ACEI/ARB use on all‐cause mortality and MACE in these patients. The results indicated that long‐term ACEI/ARB use was associated with a significant reduction in both all‐cause mortality (log‐rank *p* = 0.015; aHR, 0.44; 95% CI, 0.23–0.85; *p* = 0.014) and MACE (log‐rank *p* = 0.017; aHR, 0.58; 95% CI, 0.36–0.92; *p* = 0.021) in the myocardial injury group (Figure 5A,B). In patients without myocardial injury, ACEI/ARB use was significantly associated with reduced MACE (log‐rank *p* = 0.028; aHR, 0.49; 95% CI, 0.25–0.94; *p* = 0.032), but not with all‐cause mortality (log‐rank *p* = 0.155; aHR, 0.64; 95% CI, 0.35–1.19; *p* = 0.160) (Figure 5A,B). These findings suggest ACEI/ARB treatment leads to beneficial effects in the post‐discharge period for patients with myocardial injury.

**FIGURE 5:**
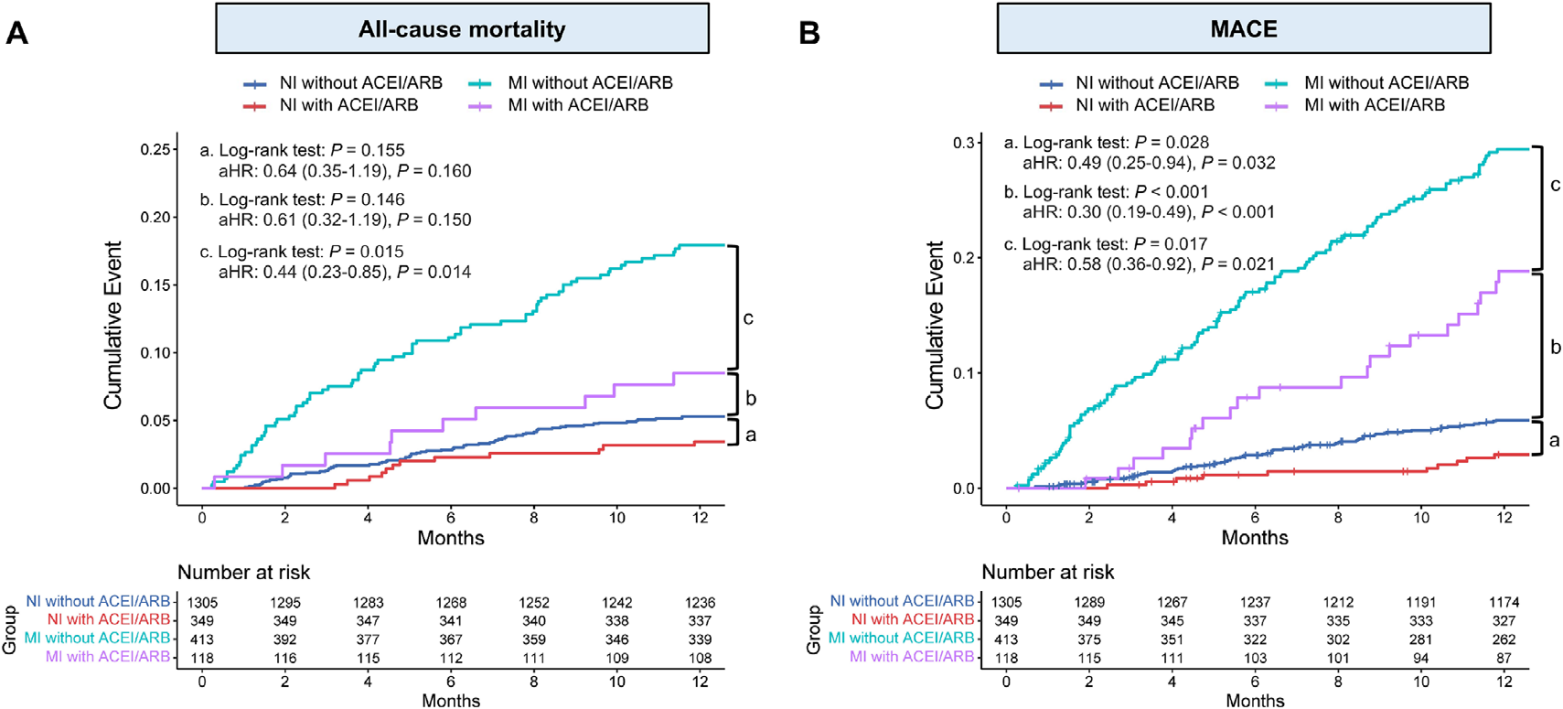
Kaplan–Meier curves for 1‐year clinical outcomes in COVID‐19 patients with and without ACEI/ARB use, stratified by myocardial injury status. (A) All‐cause mortality. (B) Major adverse cardiovascular events (MACE). ACEI/ARB, angiotensin‐converting enzyme inhibitors/angiotensin II receptor blockers; MI, myocardial injury; NI, no injury.

### 2.5 Symptoms and Health‐Related Quality of Life in Survivors

To assess the symptoms and overall quality of life 1 year after discharge, we conducted a structured follow‐up using a comprehensive questionnaire among survivors from both the myocardial injury and no injury groups. The follow‐up included evaluations of sequelae symptoms, the modified British Medical Research Council (mMRC) score, the EuroQol five‐dimension five‐level (EQ‐5D‐5L) questionnaire, and the EuroQol Visual Analogue Scale (EQ‐VAS). All surviving patients who completed the 1‐year follow‐up (*n* = 2020) responded to the electronic questionnaire. Initially, baseline characteristics of the surviving patients were compared between the myocardial injury (*n* = 447) and no injury (*n* = 1573) groups. A new 1:1 PSM was subsequently performed, yielding matched cohorts of 427 patients each (Tables S3 and S4).

Our findings revealed that 71.5% of all survivors reported experiencing at least one persistent symptom post‐discharge, with fatigue or muscle weakness being the most prevalent (25.3%), followed by sleep difficulties (16.4%) (Table 2). Both before and after PSM, survivors in the myocardial injury group exhibited significantly higher rates of sequelae symptoms compared to the no injury group (*p* < 0.05). Specifically, symptoms such as fatigue or muscle weakness, chest pain, and myalgia were more prevalent in the myocardial injury group in both unmatched and matched analyses (*p* < 0.05). Although sleep difficulties did not show a significant difference between the groups prior to PSM, they became notably more common in the myocardial injury group post‐PSM. Palpitations showed a significant difference only before matching. The mMRC dyspnea scale results indicated that a higher proportion of survivors in the myocardial injury group had scores of 1 or greater, a finding consistent both before and after matching, highlighting a greater degree of respiratory difficulty in this group. In terms of quality of life, the EQ‐5D‐5L questionnaire demonstrated that, both before and after PSM, the myocardial injury group reported more issues with pain or discomfort and anxiety or depression compared to their no injury group counterparts. The EQ‐VAS assessment further reinforced these findings, showing that myocardial injury survivors had notably lower quality of life scores (median score of 80, IQR, 75–85) compared to the no injury group (median score of 85, IQR, 75–90) both before and after matching. These results collectively suggest that 1 year after discharge, survivors in the myocardial injury group experience a higher burden of sequelae symptoms and a decreased quality of life compared to those in the no injury group, underscoring the long‐term impact of myocardial injury in COVID‐19 patients.

**TABLE 2:** Sequelae symptom and health‐related quality of life among surviving COVID‐19 patients in myocardial injury group and no injury group at 12‐month follow‐up.

		Unmatched			PSM (1:1)		
	All patients (*n* = 2020)	No injury group (*n* = 1573)	Myocardial injury group (*n* = 447)	*p*	No injury group (*n* = 427)	Myocardial injury group (*n* = 427)	*p*
Sequelae symptom							
Any one of the following symptoms, *n* (%)	1445 (71.5)	1102 (70.1)	343 (76.7)	0.006	297 (69.6)	326 (76.3)	0.025
Fatigue or muscle weakness, *n* (%)	512 (25.3)	373 (23.7)	139 (31.1)	0.002	94 (22.0)	133 (31.1)	0.003
Sleep difficulties, *n* (%)	332 (16.4)	249 (15.8)	83 (18.6)	0.168	55 (12.9)	76 (17.8)	0.046
Hair loss, *n* (%)	198 (9.8)	151 (9.6)	47 (10.5)	0.566	49 (11.5)	44 (10.3)	0.583
Smell disorder, *n* (%)	157 (7.8)	123 (7.8)	34 (7.6)	0.882	39 (9.1)	34 (8.0)	0.541
Palpitations, *n* (%)	204 (10.1)	146 (9.3)	58 (13.0)	0.022	46 (10.8)	57 (13.3)	0.248
Joint pain, *n* (%)	224 (11.1)	176 (11.2)	48 (10.7)	0.789	48 (11.2)	42 (9.8)	0.504
Decreased appetite, *n* (%)	112 (5.5)	87 (5.5)	25 (5.6)	0.960	22 (5.2)	23 (5.4)	0.878
Taste disorder, *n* (%)	112 (6.0)	94 (6.0)	28 (6.3)	0.821	23 (5.4)	27 (6.3)	0.560
Dizziness, *n* (%)	162 (8.0)	120 (7.6)	42 (9.4)	0.225	32 (7.5)	40 (9.4)	0.324
Nausea or vomiting, *n* (%)	34 (1.7)	25 (1.6)	9 (2.0)	0.538	8 (1.9)	9 (2.1)	0.806
Chest pain, *n* (%)	143 (7.1)	99 (6.3)	44 (9.8)	0.010	25 (5.9)	44 (10.3)	0.017
Sore throat or difficult to swallow, *n* (%)	54 (2.7)	42 (2.7)	12 (2.7)	0.987	8 (1.9)	12 (2.8)	0.365
Skin rash, *n* (%)	79 (3.9)	60 (3.8)	19 (4.3)	0.675	21 (4.9)	19 (4.4)	0.746
Myalgia, *n* (%)	98 (4.9)	65 (4.1)	33 (7.4)	0.005	17 (4.0)	31 (7.3)	0.038
Headache, *n* (%)	59 (2.9)	48 (3.1)	11 (2.5)	0.513	20 (4.7)	10 (2.3)	0.063
mMRC score							
0, *n* (%)	1480 (73.3)	1172 (74.5)	308 (68.9)	0.018	327 (76.6)	294 (68.9)	0.011
≥ 1, *n* (%)	540 (26.7)	401 (25.5)	139 (31.1)	0.018	100 (23.4)	133 (31.1)	0.011
EQ‐5D‐5L questionnaire							
Mobility: problems with walking around, *n* (%)	113 (5.6)	86 (5.5)	27 (6.0)	0.642	23 (5.4)	26 (6.1)	0.659
Personal care: problems with washing or dishing, *n* (%)	51 (2.5)	38 (2.4)	13 (2.9)	0.558	8 (1.9)	12 (2.8)	0.365
Usual activity: problems with usual activity, *n* (%)	41 (2.0)	31 (2.0)	10 (2.2)	0.725	15 (3.5)	9 (2.1)	0.214
Pain or discomfort, *n* (%)	533 (26.4)	394 (25.0)	139 (31.1)	0.010	107 (25.1)	134 (31.4)	0.040
Anxiety or depression, *n* (%)	453 (22.4)	327 (20.8)	126 (28.2)	0.001	93 (21.8)	119 (27.9)	0.039
EQ‐VAS							
Quality of life, median (IQR)	85 (75‐90)	85 (75‐90)	80 (75‐85)	< 0.001	85 (75‐90)	80 (75‐85)	<0.001

Abbreviations: EQ‐5D‐5L questionnaire, EuroQol five‐dimension five‐level questionnaire; EQ‐VAS, EuroQol Visual Analogue Scale; IQR, interquartile range; mMRC score, modified British Medical Research Council score; PSM, propensity score matching.

## 3 Discussion

To our knowledge, this is the largest prospective cohort study to date examining 1‐year post‐discharge outcomes in COVID‐19 survivors with versus without myocardial injury during China's Omicron wave. Our findings provide critical insights into the long‐term clinical trajectory, persistent symptom burden, and health‐related quality of life in this post‐discharge population. While our prior work established a high prevalence (30.8%) and substantial in‐hospital mortality risk associated with myocardial injury during the acute Omicron wave, the current study extends these observations by demonstrating that the elevated risk persists well beyond hospital discharge. Specifically, patients with myocardial injury experienced significantly higher 1‐year all‐cause mortality and MACE compared with those without injury, with consistent effects observed across diverse prespecified subgroups. Notably, long‐term ACEI/ARB use was associated with significantly reduced all‐cause mortality and MACE in the myocardial injury group. Furthermore, 71.5% of patients reported at least one persistent symptom at 1‐year post‐discharge—most commonly fatigue or muscle weakness and sleep difficulties—and those with myocardial injury exhibited a greater symptom burden and a marked deterioration in quality of life relative to their non‐injured counterparts. Our study extends the evidence base as an Omicron‐era cohort in China, combining clinical outcomes with quality‐of‐life assessment and providing the first ACEI/ARB association analysis in myocardial injury survivors.

Our results underscore that myocardial injury associated with SARS‐CoV‐2 infection is an independent risk factor for post‐discharge all‐cause mortality and MACE among patients with COVID‐19. While some studies suggest that myocardial injury in severe COVID‐19 survivors is nonprogressive or has no long‐term impact on clinical outcomes [18, 19], more studies seem to support the association between COVID‐19‐related myocardial injury and long‐term adverse clinical outcomes [20, 21, 22, 23]. A prospective study from Italy demonstrated that myocardial injury during hospitalization in COVID‐19 survivors could predict a higher risk of mortality (HR, 1.95; 95% CI, 1.05−3.61) and cardiovascular sequelae [21]. Conversely, a recent large cohort study from Singapore during Omicron predominance found no increased overall cardiovascular risk in COVID‐19 survivors (aHR, 1.01; 95% CI, 0.97–1.07), though elevated risks were observed in hospitalized and severe cases [24]. Furthermore, a follow‐up study involving 172 COVID‐19 patients also indicated that myocardial injury was associated with adverse clinical outcomes within 1 year, independent of age and gender [22]. Additionally, a prospective multicenter study utilizing cardiac magnetic resonance imaging (CMR) for follow‐up has found that up to 42% of patients with moderate to severe COVID‐19 continue to exhibit myocardial injury 3 months after discharge, indicating that the impact of myocardial injury may be persistent in some individuals [25]. Evidence from induced pluripotent stem cell research suggests that CCL2‐mediated endothelial injury can cause persistent cardiac dysfunction by inducing oxidative stress and post‐translational modifications of cardiac proteins [26]. Our finding highlighted the important role that myocardial injury played in influencing long‐term outcomes, suggesting that it might serve as a key prognostic indicator in patients with COVID‐19, especially during the Omicron pandemic.

The strong predictive capability of hs‐cTnI levels for clinical outcomes in COVID‐19 patients further supported the utility of this biomarker in risk stratification [27, 28, 29]. Troponin elevation in COVID‐19 patients may be closely associated with acute myocarditis patterns [30]. In patients with myocardial injury, those with relatively higher levels of hs‐cTnI and high‐sensitivity C‐reactive protein at admission appeared more likely to experience cardiac involvement during 6‐month follow‐up [31]. Our successive studies found that elevated hs‐cTnI not only reflected the extent of cardiac injury during the acute phase [11, 12], but also signaled an increased susceptibility to adverse events in the post‐acute phase. When evaluating the cardiac health of COVID‐19 patients, special attention should be given to those with hs‐cTnI levels slightly below the upper normal limit. Although these levels do not exceed the normal range, even a slight increase may indicate subtle myocardial injury [11]. Despite not reaching the traditional threshold for diagnosing myocardial damage, minor elevations in troponin levels can still be associated with adverse clinical outcomes [11]. These insights emphasize the need for vigilant monitoring and tailored therapeutic strategies for patients with myocardial injury, aiming to mitigate long‐term risks and improve overall survival and quality of life [32]. The mechanisms underlying myocardial injury during the acute phase of COVID‐19 infection are gradually being elucidated, including factors such as direct viral invasion, systemic inflammatory response, cytokine storm, hypoxic injury, microvascular dysfunction, hypercoagulability, and endothelial dysfunction [33, 34].

The persistence of adverse outcomes may be explained by several interrelated mechanisms extending beyond the acute phase. First, chronic low‐grade inflammation—driven by persistently elevated cytokines (IL‐1β, IL‐6, TNF‐α)—promotes fibroblast activation and myocardial fibrosis [35]. Second, endothelial dysfunction and microvascular injury, resulting from SARS‐CoV‐2 infection of endothelial cells via ACE2, impair perfusion and contribute to ongoing ischemia and exertional symptoms [36]. Third, mitochondrial injury leads to energy depletion and contractile dysfunction [37, 38]. Finally, persistent viral antigen or RNA in cardiac tissue may drive chronic immune activation [39]. Notably, elevated hs‐cTnI reflects ongoing subclinical myocardial damage driven by these chronic processes, collectively contributing to the increased long‐term mortality, MACE, and symptom burden observed in our study.

One notable finding is the beneficial effect of the long‐term use of ACEI/ARB in reducing both all‐cause mortality and MACE in the myocardial injury group. This suggested a protective role for these medications, likely due to their ability to mitigate adverse cardiac remodeling, reduce afterload, and improve endothelial function [17]. Additionally, their anti‐inflammatory effects and potential to attenuate the dysregulation of renin‐angiotensin‐aldosterone system (exacerbated in conditions of myocardial injury) could contribute to improved long‐term outcomes [17].Multiple meta‐analyses have also demonstrated the safety and efficacy of ACEI/ARB in COVID‐19 patients [40, 41]. In fact, for COVID‐19 patients taking ACEI/ARB, these medications should be withdrawn in rare cases [42]. In SARS‐CoV‐2‐induced cardiomyocyte injury, treatment with angiotensin II (AngII) has been shown to decrease contractility, leading to earlier onset of contractile dysfunction and a significant elevation in inflammatory cytokines, which indicates that blocking Ang II may offer a protective effect against SARS‐CoV‐2‐related myocardial injury [43]. Captopril can alleviate the inflammatory response and apoptosis in cardiomyocytes induced by SARS‐CoV‐2 by attenuating the TNF signaling pathway [44]. Several methodological considerations should be noted when interpreting these findings. The observational nature of the ACEI/ARB analysis may introduce immortal time bias, confounding by indication, and lack of continuous adherence monitoring. While we adjusted for measured confounders using PSM and multivariable Cox regression, residual confounding cannot be excluded. Therefore, these findings should be interpreted as associative rather than causal, and warrant confirmation in future randomized controlled trials.

Furthermore, our follow‐up of survivors 1 year after discharge indicated a high prevalence of at least one persistent symptom, with fatigue or muscle weakness and sleep difficulties being the most common. These sequelae were more pronounced in the myocardial injury group, who also reported a significantly reduced quality of life. Although questionnaires assessing symptoms and quality of life in COVID‐19 patients have been previously reported [45], our study placed a greater emphasis on comparing these aspects between patients with and without myocardial injury during the Omicron outbreak in China. Previous studies have also indicated that COVID‐19‐related myocardial injury is associated with increased symptoms and a decline in quality of life [46, 47]; however, these studies typically involved shorter follow‐up periods of less than 6 months. Our results additionally revealed that COVID‐19 patients in the myocardial injury group exhibited a higher prevalence of anxiety or depression 1 year after discharge, suggesting a potential association between myocardial injury and psychological symptoms in COVID‐19 patients. These follow‐up findings were consistent with the emerging understanding of long COVID, emphasizing the need for comprehensive rehabilitation programs tailored to address these lingering effects. These results also underscored the necessity for targeted post‐discharge interventions and highlighted the importance of ongoing cardiovascular monitoring in the long‐term care of COVID‐19 survivors.

This study has several limitations that warrant consideration. First, it is a single‐center study conducted in China, which, despite having a relatively large sample size, may limit the generalizability of our findings to other populations and healthcare settings. Second, although we used PSM and multivariable Cox regression to control for measured confounders—including the baseline imbalance in age and comorbidities between groups—the possibility of residual confounding by unmeasured factors cannot be entirely excluded. Third, data collection relied on telephone follow‐up, which precluded the acquisition of clinical examinations or laboratory tests during the 12‐month post‐discharge period. This approach may have introduced recall bias and limited the depth of clinical insight. ACEI/ARB use was assessed at a single time point, without documentation of treatment duration, adherence, initiation timing, or medication changes; this limits causal interpretation, together with the potential for immortal time bias and confounding by indication. Additionally, serial hs‐cTnI measurements were not available for all patients, preventing systematic distinction between acute and chronic myocardial injury. These limitations constrained our ability to fully characterize myocardial injury progression and its long‐term trajectory. Finally, vaccination status was unavailable and constitutes an important unmeasured confounder. Since unvaccinated individuals faced a higher risk of severe infection and myocardial injury during the Omicron wave, they were likely overrepresented in the myocardial injury group, which may have led to an overestimation of the observed association between myocardial injury and adverse outcomes. Although we adjusted for measured confounders using PSM and multivariable regression, residual confounding cannot be ruled out.

## 4 Conclusion

Overall, this study reveals for the first time in China's Omicron wave that myocardial injury during hospitalization is associated with significantly increased 1‐year mortality and MACE among discharged survivors, with persistent symptoms and reduced quality of life. The protective association of ACEI/ARB use in this population underscores a clinically actionable intervention for long‐term management. Future research should focus on elucidating the underlying mechanisms driving these persistent effects and developing therapeutic strategies to mitigate them.

## 5 Materials and Methods

### 5.1 Study Design and Patient Information

This prospective cohort study was conducted at Tongji Hospital (Wuhan, China), focusing on patients hospitalized with COVID‐19, focusing on the inpatients with COVID‐19 during the Omicron pandemic. Laboratory confirmation of SARS‐CoV‐2 infection was performed by two consecutive positive results from high‐throughput sequencing or real‐time reverse transcriptase–polymerase chain reaction assays of nasal and pharyngeal swabs. This study included 2378 surviving COVID‐19 patients discharged from December 2022 to March 2023. All of them received hs‐cTnI detection during hospitalization. All discharged patients met the standardized discharge criteria outlined in the “Chinese clinical guidance for COVID‐19 pneumonia diagnosis and treatment” issued by the National Health Commission of China. Patients were stratified into myocardial injury group and no injury groups based on the presence or absence of myocardial injury during hospitalization. Myocardial injury was defined as at least one hs‐cTnI level exceeding the 99th percentile upper reference limit (> 26.2 pg/mL) during the hospital stay, with a subsequent rise and/or fall of hs‐cTnI values consistent with acute myocardial injury per the Fourth Universal Definition of Myocardial Infarction. hs‐cTnI was measured using the STAT High Sensitive Troponin‐I Reagent Kit (3P25‐77, Abbott, Dublin, Ireland), with the 99th percentile upper reference limit of 26.2 pg/mL as defined by the manufacturer. Patients were divided into myocardial injury and no injury groups based on this threshold.

The study was conducted in accordance with the principles of the Declaration of Helsinki and approved by the Research Ethics Committee of Tongji Hospital (no. TJ‐IRB20210138). Informed consent for participation in the study was obtained from all patients. During the follow‐up process, the study objectives, procedures, potential risks, and benefits were thoroughly explained to each participant. All patients were given the opportunity to ask questions and were assured that their participation was voluntary and that they could withdraw from the study at any time without any impact on their standard medical care.

### 5.2 Data Collection and Follow‐Up

Demographic and clinical data for all COVID‐19 patients were extracted from the electronic medical record database of Tongji Hospital. The data collected included age, gender, smoking and alcohol consumption history, and comorbidities, including hypertension, coronary artery disease, chronic heart failure, diabetes, chronic kidney disease, and solid tumors. Basic vital signs, including temperature, respiratory rate, pulse rate, diastolic, and systolic blood pressure, were also recorded, along with clinical laboratory parameters such as hs‐cTnI, routine blood test results, blood biochemistry, and coagulation function. To ensure the accuracy of baseline information, three independent researchers reviewed all original data.

A team of trained physicians conducted a 1‐year telephone follow‐up using a standardized questionnaire. For all potential endpoint events reported, medical records (including discharge summaries, death certificates, and imaging reports) were obtained from the respective hospitals and reviewed by an independent Clinical Events Committee consisting of three cardiologists blinded to myocardial injury status. All reported deaths were verified through death certificates or hospital records. All adjudications required consensus between at least two committee members. The follow‐up primarily focused on outcome events and long‐term medication use (defined as patient‐reported use at the 12‐month follow‐up, reflecting chronic post‐discharge therapy rather than acute in‐hospital exposure). The primary outcome was calculated for all COVID‐19 patients 1 year after their initial discharge, including all‐cause mortality and MACE. MACE was defined as a composite of the following events: (1) cardiovascular death: defined as death resulting from acute coronary syndrome, cardiac rupture, severe arrhythmias, refractory heart failure, stroke, cardiovascular procedures, cardiovascular bleeding, or other definite cardiovascular causes, or death of unknown cause that was adjudicated by the Clinical Events Committee as probably due to cardiovascular causes; (2) nonfatal myocardial infarction: defined according to the Fourth Universal Definition of Myocardial Infarction, including a rise and/or fall of cardiac biomarkers with at least one value above the 99th percentile upper reference limit, accompanied by clinical evidence of myocardial ischemia; (3) nonfatal stroke: defined as a focal or global neurological deficit attributed to a cerebrovascular cause, lasting more than 24 h, confirmed by neuroimaging, and excluding other causes; and (4) hospitalization for heart failure: defined as hospital admission with a primary diagnosis of heart failure, length of stay ≥ 24 h, with documented new or worsening heart failure symptoms and objective evidence, and receiving intensification of heart failure‐specific therapy.

For patients who survived after 1 year of follow‐up, an electronic questionnaire was administered, including a self‐reported symptom questionnaire, the mMRC dyspnea scale, the EQ‐5D‐5L questionnaire, and the EQ‐VAS. All instruments used were validated Chinese versions. The questionnaire was self‐administered at 12 months post‐discharge. Questionnaire analysts were blinded to myocardial injury status throughout data processing and analysis. The mMRC scale is a five‐category scale used to characterize the degree of dyspnea induced by physical activity, where higher scores indicate more severe dyspnea [48]. The EQ‐5D‐5L questionnaire is a validated tool for assessing quality of life by evaluating five dimensions: mobility, self‐care, usual activities, pain or discomfort, and anxiety or depression, each with five levels ranging from no problems to extreme problems [49]. The EQ‐VAS provides a subjective assessment of general health status, ranging from 0 (worst possible quality of life) to 100 (best possible quality of life), with higher scores reflecting better perceived health [50]. The telephone follow‐ups were independently conducted by three researchers, and all results were reviewed and verified by another researcher to ensure data integrity. To ensure blinding, the follow‐up interviewers, the Clinical Events Committee members, and the statisticians were all blinded to patients' myocardial injury status until database lock.

### 5.3 Statistical Analysis and Mapping

Statistical analyses and visualization were conducted using SPSS (version 24.0; IBM, Armonk, NY, USA), R (version 4.1.1; R Foundation for Statistical Computing, Vienna, Austria), and GraphPad Prism (version 9.0; GraphPad Software, San Diego, CA, USA). To minimize the impact of confounding variables, we employed a 1:1 PSM analysis within the R environment (version 4.1.1; R Foundation for Statistical Computing, Vienna, Austria) using the MatchIt package (version 4.5.0). Propensity scores were estimated using a multivariable logistic regression model including the following prespecified baseline covariates: age, gender, comorbidities (hypertension, coronary artery disease, chronic heart failure, type 2 diabetes, chronic kidney disease, and solid tumors), vital signs (temperature, respiratory rate, pulse rate, systolic and diastolic blood pressure), and clinical laboratory parameters (routine blood tests, blood biochemistry, and coagulation function). Nearest‐neighbor matching was performed with a fixed 1:1 matching ratio, a caliper width of 0.05 (standard deviation of the logit‐transformed propensity score), and without replacement. Covariate balance between the matched groups was assessed using standardized mean differences (SMD), with an SMD < 0.1 considered indicative of acceptable balance. For Cox proportional hazards regression, variables were selected for adjustment based on clinical relevance and baseline imbalance identified before PSM. In the primary analysis, the following covariates were included in the multivariable model: age, gender, hypertension, coronary artery disease, chronic heart failure, chronic kidney disease, and hs‐cTnI group (myocardial injury vs. no injury). All continuous variables satisfied the linearity assumption, assessed by martingale residuals. The proportional hazards assumption was formally tested using Schoenfeld residuals. Results are presented as aHR with 95% CIs. Subgroup analyses were prespecified based on clinical relevance and prior literature [11, 27, 51], including age, gender, smoking, hypertension, coronary artery disease, chronic heart failure, type 2 diabetes, ALT, eGFR, and D‐dimer. Interaction *p*‐values were calculated for each subgroup to assess effect heterogeneity.

The distribution and homoscedasticity of each dataset were tested using D'Agostino's and Pearson's omnibus normality tests. Normally distributed continuous data were reported as means (standard deviation) and compared using Student's *t*‐test. Non‐normally distributed continuous data were reported as medians (IQR) and compared using the Wilcoxon rank‐sum test. Categorical data were presented as counts (percentages) and compared using the chi‐square test, Fisher's exact test, or Cochran–Mantel–Haenszel test, as appropriate.

The proportional hazards (PH) assumption for all Cox regression models was assessed using Schoenfeld residual‐based tests. A global test was performed for each model, and *p* > 0.05 was considered to indicate no violation of the PH assumption. Additionally, scaled Schoenfeld residual plots were visually inspected for each covariate to confirm the absence of time‐varying effects. The PH assumption was satisfied for all covariates in all models (global test *p* > 0.05 for all models).

Survival analysis methods, including Kaplan–Meier curves and Cox proportional hazards models, were used to compare time‐to‐event outcomes between the myocardial injury and no injury groups, as well as among various subgroups. To assess the dose‐response relationship beyond the binary threshold, we additionally performed quartile‐based survival analyses and RCS regression. Nonlinearity was tested using the likelihood ratio test. All tests were two‐sided, and statistical significance was set at *p* < 0.05. Subgroup and symptom comparisons were exploratory in nature; therefore, no adjustment for multiple comparisons was applied. *p* values near 0.05 were interpreted conservatively. No missing data were present for any of the variables included in the final analysis.

## Author Contributions

*Conceptualization*: W.H. and D.W.W. designed the study and developed the research framework. *Data collection*: W.H., Q.H., K.X., G.L., Y.Y., and J.W. conducted data collection and patient follow‐up. *Methodology*: Q.H., G.L., B.Y., Y.S., H.W., and W.H. were responsible for developing the methodology and analytical approach, including statistical analyses. *Data Analysis*: W.H. and Q.H. performed the data analysis and interpreted the results. *Writing – original draft*: W.H., Q.H., and D.W.W. drafted the initial manuscript. *Writing – review and editing*: All authors contributed to the critical revision of the manuscript for important intellectual content. *Supervision*: D.W.W. provided oversight and guidance throughout the study process. All authors have read and approved the final manuscript.

## Funding

This work was supported in part by the projects of National Natural Science Foundation of China (Nos. 82100526, 824B2007, 82241034, 82330010, and W2611115), National Key R&D Program of China (2024YFC3044500), Fundamental Research Funds for the Central Universities (YCJJ20252101), Hubei natural science foundation of China (No. 2025AFB589), and the Scientific Research Project of Hubei Province (Nos. 2024CSA067, 2025CSA042).

## Ethics Statement

The study was conducted in accordance with the principles of the Declaration of Helsinki and approved by the Research Ethics Committee of Tongji Hospital, Tongji Medical College, Huazhong University of Science and Technology (no. TJ‐IRB20210138). Informed consent for participation in the study was obtained from all patients. All participant information has been anonymized. This trial has been registered in ClinicalTrials.gov (NCT05615792).

## Conflicts of Interest

The authors declare no conflicts of interest.

## Supporting information




**Supporting File 1**: mco271119‐sup‐0001‐SupMat.docx

## Data Availability

The data underlying this article will be shared upon reasonable request by the corresponding author.
