# Supplementary material for: Long‐Term Effects of Myocardial Injury on 1‐Year Outcomes in Hospital Survivors With COVID‐19: A Prospective Cohort Study
Source: MedComm (2020). 2026 Nov 3;7(11):e71119. doi: 10.1002/mco2.71119 (PMC13732117; doi:10.1002/mco2.71119)
Supplement: Supplementary file 1 [file MCO2-7-e71119-s001.docx]

**Supplementary Material**

**Long-term effects of myocardial injury on one-year outcomes in hospital survivors with COVID-19: a prospective cohort study**

Wu He^1#^, Qiongyao He^2#^, Ke Xu^1^, Gen Li^1^, Yongcui Yan^1^, Bo Yu^1^, Yujing Su^1^, Junfang Wu^1^, Hong Wang^1^, Dao Wen Wang^1*^

1. Division of Cardiology, Department of Internal Medicine, Tongji Hospital, Tongji Medical College, Huazhong University of Science and Technology; Hubei Key Laboratory of Genetics and Molecular Mechanisms of Cardiological Disorders, Wuhan 430030, China

2. Department of Endocrinology, Department of Internal Medicine, Tongji Hospital, Tongji Medical College, Huazhong University of Science and Technology, Wuhan 430030, China

Corresponding author:

Dao Wen Wang

Division of Cardiology, Department of Internal Medicine

Tongji Hospital, Tongji Medical College

Huazhong University of Science & Technology

1095# Jiefang Ave.

Wuhan 430030, China

Tel. & Fax: 86-27-8366-3399

Email: [dwwang@tjh.tjmu.edu.cn](mailto:dwwang@tjh.tjmu.edu.cn)

^#^Wu He and Qiongyao He equally contributed to this work.

**Figure S1. Survival analysis of the components of MACE for COVID-19 patients in myocardial injury and no injury groups before and after PSM.**

(A) Cardiovascular death before PSM.

(B) Cardiovascular death after PSM.

(C) Non-fatal myocardial infarction before PSM.

(D) Non-fatal myocardial infarction after PSM.

(E) Non-fatal stroke before PSM.

(F) Non-fatal stroke after PSM.

(G) Hospitalization for heart failure before PSM.

(H) Hospitalization for heart failure after PSM.

**Figure S2. Survival analysis of one-year clinical outcomes ranked by hs-cTnI quartiles in COVID-19 patients.**

(A) The Kaplan–Meier survival curve of all-cause mortality for all COVID-19 patients.

(B) The Kaplan–Meier survival curve of MACE for all COVID-19 patients.

(C) The Kaplan–Meier survival curve of all-cause mortality for COVID-19 patients in no injury group.

(D) The Kaplan–Meier survival curve of MACE for COVID-19 patients in no injury group.

(E) The Kaplan–Meier survival curve of all-cause mortality for COVID-19 patients in myocardial injury group.

(F) The Kaplan–Meier survival curve of MACE for COVID-19 patients in myocardial injury group.

**Table S1. Detailed laboratory parameters of COVID-19 patients in myocardial injury and no injury groups.**

|  | **All patients (n=2185)** | **Unmatched** | | | **PSM (1:1)** | | |
| --- | --- | --- | --- | --- | --- | --- | --- |
|  |  | **No injury group (n=1654)** | **Myocardial injury group (n=531)** | **p** | **No injury group (n=507)** | **Myocardial injury group (n=507)** | **p** |
| Glucose, mmol/L | 6.7 (5.7-8.5) | 6.7 (5.7-8.5) | 6.7 (5.7-8.3) | 0.504 | 6.7 (5.8-8.7) | 6.7 (5.7-8.3) | 0.580 |
| HbA1c, % | 7.2 (6.7-7.9) | 7.2 (6.7-7.9) | 7.2 (6.7-7.7) | 0.256 | 7.2 (6.8-7.9) | 7.2 (6.7-7.7) | 0.353 |
| RBC count, ×10^12^/L | 3.99 (3.53-4.43) | 4.00 (3.55-4.41) | 3.96 (3.49-4.45) | 0.812 | 4.01 (3.53-4.43) | 3.96 (3.51-4.46) | 0.798 |
| Hemoglobin, g/L | 120 (103-133) | 120 (103-133) | 120 (104-134) | 0.973 | 120 (104-133) | 120 (104-133) | 0.549 |
| WBC count, ×10^9^/L | 6.10 (4.41-8.23) | 6.09 (4.47-8.27) | 6.12 (4.27-8.09) | 0.691 | 5.86 (4.24-8.20) | 6.07 (4.21-8.04) | 0.918 |
| Platelet count, ×10^9^/L | 205 (146-274) | 202 (143-269) | 215 (156-285) | 0.007 | 200 (147-267) | 213 (154-282) | 0.095 |
| ALT, U/L | 21 (14-33) | 20 (14-33) | 22 (15-36) | 0.006 | 21 (14-34) | 22 (15-35) | 0.233 |
| AST, U/L | 26 (18-38) | 25 (18-37) | 27 (19-40) | 0.059 | 25 (18-37) | 27 (19-40) | 0.169 |
| LDH, U/L | 247 (200-319) | 247 (201-323) | 248 (197-311) | 0.372 | 251 (202-324) | 248 (197-312) | 0.468 |
| Total bilirubin, umol/L | 8.4 (6.0-12.0) | 8.4 (6.0-11.9) | 8.5 (5.9-12.2) | 0.505 | 8.0 (5.7-11.8) | 8.4 (5.9-12.2) | 0.074 |
| Total protein, g/L | 66.7 (62.5-70.8) | 66.7 (62.3-70.7) | 66.8 (62.8-71.5) | 0.236 | 66.8 (62.3-71.1) | 66.8 (62.9-71.6) | 0.518 |
| Albumin/globulin | 1.11 (0.93-1.32) | 1.10 (0.93-1.32) | 1.12 (0.83-1.32) | 0.852 | 1.10 (0.94-1.29) | 1.12 (0.92-1.32) | 0.715 |
| T-Ch, mmol/L | 3.73 (3.11-4.41) | 3.73 (3.12-4.40) | 3.71 (3.06-4.44) | 0.905 | 3.75 (3.11-4.40) | 3.69 (3.06-4.44) | 0.692 |
| Triglyceride, mmol/L | 1.44 (1.12-1.86) | 1.44 (1.12-1.84) | 1.45 (1.13-1.89) | 0.438 | 1.47 (1.16-1.88) | 1.45 (1.13-1.90) | 0.983 |
| LDL, mmol/L | 2.15 (1.65-2.74) | 2.14 (1.64-2.73) | 2.18 (1.68-2.76) | 0.534 | 2.14 (1.64-2.71) | 2.18 (1.68-2.76) | 0.547 |
| HDL, mmol/L | 0.88 (0.73-1.03) | 0.89 (0.74-1.04) | 0.87 (0.70-1.02) | 0.104 | 0.89 (0.74-1.03) | 0.87 (0.70-1.02) | 0.190 |
| Creatinine, μmol/L | 80 (64-107) | 80 (64-110) | 78 (63-100) | 0.115 | 79 (63-114) | 78 (64-100) | 0.416 |
| BUN, mmol/L | 6.3 (4.7-9.0) | 6.4 (4.7-9.1) | 6.0 (4.5-8.8) | 0.019 | 6.4 (4.6-9.2) | 6.0 (4.5-8.8) | 0.094 |
| eGFR, mL/min/1.73m^2 | 80.5 (55.2-94.2) | 79.7 (52.8-93.7) | 81.9 (59.9-94.6) | 0.083 | 81.5 (51.7-94.9) | 81.8 (59.8-94.5) | 0.382 |
| Sodium, mmol/L | 137.7 (135.1-140.1) | 137.7 (135.1-140.1) | 137.8 (135.2-140.0) | 0.888 | 137.5 (134.9-139.9) | 137.8 (135.2-140.1) | 0.390 |
| Potassium, mmol/L | 4.09 (3.78-4.45) | 4.10 (3.79-4.46) | 4.07 (3.74-4.40) | 0.207 | 4.14 (3.77-4.52) | 4.07 (3.73-4.41) | 0.064 |
| APTT | 36.1 (32.7-40.0) | 36.1 (32.7-39.9) | 35.8 (32.6-40.5) | 0.992 | 35.9 (32.5-39.9) | 35.9 (32.7-40.5) | 0.431 |
| Prothrombin time, s | 13.3 (12.5-14.1) | 13.3 (12.5-14.1) | 13.2 (12.4-14.1) | 0.583 | 13.2 (12.5-14.1) | 13.2 (12.4-14.1) | 0.890 |
| Thrombin time, s | 17.6 (16.4-18.8) | 17.6 (16.4-18.8) | 17.6 (16.3-18.8) | 0.661 | 17.6 (16.2-18.9) | 17.7 (16.4-18.8) | 0.916 |
| International normalized ratio | 1.03 (0.97-1.11) | 1.03 (0.97-1.11) | 1.03 (0.97-1.11) | 0.803 | 1.02 (0.96-1.11) | 1.03 (0.97-1.11) | 0.735 |
| Fibrinogen, mg/L | 4.31 (3.52-5.26) | 4.33 (3.52-5.24) | 4.24 (3.52-5.29) | 0.464 | 4.37 (3.55-5.24) | 4.22 (3.42-5.28) | 0.172 |
| D-dimer, ug/mL FEU | 0.89 (0.50-1.79) | 0.89 (0.49-1.81) | 0.90 (0.52-1.72) | 0.988 | 0.88 (0.50-1.88) | 0.90 (0.53-1.72) | 0.923 |

Data are presented as median (IQR). Keys: HbA1c, glycated hemoglobin; RBC, red blood cell; WBC, white blood cell; ALT, alanine aminotransferase; AST, aspartate aminotransferase; LDH, lactate dehydrogenase; LDL, low-density lipoprotein; HDL, high-density lipoprotein; BUN, blood urea nitrogen; eGFR, estimated glomerular filtration rate; APTT, activated partial thromboplastin time; INR, international normalized ratio.

**Table S2. Long-term medication use of COVID-19 patients in myocardial injury group and no injury group during one-year follow-up.**

|  | **All patients (n=2185)** | **Unmatched** | | | **PSM (1:1)** | | |
| --- | --- | --- | --- | --- | --- | --- | --- |
|  |  | **No injury group (n=1654)** | **Myocardial injury group (n=531)** | **p** | **No injury group (n=507)** | **Myocardial injury group (n=507)** | **p** |
| ACEI/ARB, n (%) | 467 (21.4) | 349 (21.1) | 118 (22.2) | 0.583 | 123 (24.3) | 109 (21.5) | 0.295 |
| Diuretic, n (%) | 459 (21.0) | 360 (21.8) | 99 (18.6) | 0.124 | 109 (21.5) | 95 (18.7) | 0.273 |
| CCB, n (%) | 665 (30.0) | 506 (30.6) | 149 (28.1) | 0.268 | 165 (32.5) | 146 (28.8) | 0.196 |
| β-blocker, n (%) | 433 (19.8) | 324 (19.6) | 109 (20.5) | 0.637 | 105 (20.7) | 106 (20.9) | 0.938 |
| Statins, n (%) | 524 (24.0) | 403 (24.4) | 121 (22.8) | 0.459 | 135 (26.6) | 114 (22.5) | 0.125 |
| Metformin, n (%) | 160 (7.3) | 126 (7.6) | 34 (6.4) | 0.350 | 42 (8.3) | 32 (6.3) | 0.227 |
| Nitrates, n (%) | 159 (7.3) | 122 (7.4) | 37 (7.0) | 0.753 | 32 (6.3) | 35 (6.9) | 0.705 |
| Anticoagulant drugs, n (%) | 79 (3.6) | 58 (3.5) | 21 (4.0) | 0.630 | 19 (3.7) | 21 (4.1) | 0.747 |
| Antiplatelet drugs, n (%) | 411 (18.8) | 309 (18.7) | 102 (19.2) | 0.787 | 105 (20.7) | 96 (18.9) | 0.478 |

Keys: PSM, propensity score matching; ACEI/ARB, angiotensin-converting enzyme inhibitors/angiotensin II receptor blockers; CCB, Calcium channel blockers.

**Table S3. Baseline of surviving COVID-19 patients in myocardial injury group and no injury group.**

|  | **All patients (n=2020)** | **Unmatched** | | | **PSM (1:1)** | | |
| --- | --- | --- | --- | --- | --- | --- | --- |
|  |  | **No injury group (n=1573)** | **Myocardial injury group (n=447)** | **p** | **No injury group (n=427)** | **Myocardial injury group (n=427)** | **p** |
| Gender: Male, n (%) | 1155 (58.5) | 897 (57.0) | 285 (63.8) | 0.011 | 273 (63.9) | 276 (64.6) | 0.830 |
| Age, years | 67 (56-75) | 66 (55-74) | 71 (59-80) | <0.001 | 71 (59-79) | 71 (59-79) | 0.997 |
| Age≥70 years, n (%) | 860 (42.6) | 620 (39.4) | 240 (53.7) | <0.001 | 223 (52.2) | 223 (52.2) | >0.999 |
| Age<70 years, n (%) | 1160 (57.4) | 953 (60.6) | 207 (46.3) | <0.001 | 204 (47.8) | 204 (47.8) | >0.999 |
| Smoking, n (%) | 342 (16.9) | 268 (17.0) | 74 (16.6) | 0.810 | 71 (16.6) | 71 (16.6) | >0.999 |
| Drinking, n (%) | 205 (10.1) | 165 (10.5) | 40 (8.9) | 0.341 | 41 (9.6) | 39 (9.1) | 0.814 |
| Hypertension, n (%) | 708 (35.0) | 548 (34.8) | 160 (35.8) | 0.708 | 137 (32.1) | 155 (36.3) | 0.194 |
| CHD, n (%) | 263 (13.0) | 192 (12.2) | 71 (15.9) | 0.041 | 59 (13.8) | 62 (14.5) | 0.768 |
| CHF, n (%) | 140 (6.9) | 97 (6.2) | 43 (9.6) | 0.011 | 37 (8.7) | 36 (8.4) | 0.903 |
| T2DM, n (%) | 416 (20.6) | 329 (20.9) | 87 (19.5) | 0.503 | 97 (22.7) | 83 (19.4) | 0.240 |
| CKD, n (%) | 267 (13.2) | 187 (11.9) | 80 (17.9) | 0.001 | 69 (16.2) | 74 (17.3) | 0.647 |
| Solid tumors, n (%) | 160 (7.9) | 122 (7.8) | 38 (8.5) | 0.607 | 25 (5.9) | 35 (8.2) | 0.181 |
| Temperature, °C | 36.5 (36.4-36.7) | 36.5 (36.4-36.7) | 36.5 (36.4-36.7) | 0.998 | 36.5 (36.3-36.8) | 36.5 (36.4-36.7) | 0.592 |
| RR, breaths/min | 20 (20-20) | 20 (20-20) | 20 (20-20) | 0.251 | 20 (20-20) | 20 (20-20) | 0.455 |
| Pulse, beats/min | 84 (78-92) | 84 (78-92) | 83 (78-91) | 0.546 | 83 (78-92) | 83 (78-91) | 0.985 |
| SBP, mmHg | 126 (118-137) | 126 (117-137) | 127 (119-139) | 0.323 | 127 (118-138) | 126 (118-139) | 0.938 |
| DBP, mmHg | 78 (71-84) | 78 (71-84) | 78 (72-85) | 0.984 | 78 (70-84) | 78 (72-85) | 0.932 |
| **Laboratory results** |  |  |  |  |  |  |  |
| hs-cTnI, pg/mL | 6.6 (2.5-21.4) | 4.4 (2.0-8.9) | 44.7 (32.4-97.4) | <0.001 | 2.1 (1.9-4.9) | 44.0 (32.2-99.5) | <0.001 |
| Glucose, mmol/L | 6.7 (5.7-8.5) | 6.7 (5.7-8.5) | 6.8 (5.7-8.5) | 0.945 | 6.7 (5.8-8.7) | 6.8 (5.7-8.5) | 0.935 |
| HbA1c, % | 7.2 (6.7-7.9) | 7.2 (6.7-7.9) | 7.2 (6.7-7.7) | 0.363 | 7.2 (6.7-7.9) | 6.7 (6.7-7.7) | 0.736 |
| RBC count, ×10^12^/L | 3.99 (3.52-4.43) | 4.00 (3.54-4.42) | 3.95 (3.46-4.45) | 0.624 | 4.02 (3.56-4.45) | 3.96 (3.46-4.47) | 0.393 |
| Hemoglobin, g/L | 120 (103-133) | 120 (103-133) | 119 (103-133) | 0.585 | 121 (106-134) | 119 (103-133) | 0.160 |
| WBC count, ×10^9^/L | 6.10 (4.46-8.25) | 6.10 (4.50-8.27) | 6.10 (4.34-8.22) | 0.765 | 5.87 (4.30-8.17) | 6.03 (4.25-8.20) | 0.896 |
| Platelet count, ×10^9^/L | 205 (145-272) | 203 (143-268) | 216 (155-282) | 0.022 | 201 (148-263) | 214 (154-280) | 0.104 |
| ALT, U/L | 20 (14-33) | 20 (14-33) | 22 (15-36) | 0.008 | 20 (14-33) | 22 (15-34) | 0.244 |
| AST, U/L | 26 (18-38) | 25 (18-37) | 27 (19-40) | 0.040 | 25 (18-37) | 27 (19-40) | 0.141 |
| LDH, U/L | 247 (200-322) | 246 (201-325) | 252 (198-318) | 0.824 | 244 (201-319) | 253 (199-318) | 0.624 |
| Total bilirubin, umol/L | 8.5 (6.0-12.0) | 8.4 (6.1-11.9) | 8.5 (5.8-12.2) | 0.613 | 8.1 (5.8-11.9) | 8.4 (5.9-12.5) | 0.172 |
| Total protein, g/L | 66.7 (62.4-70.8) | 66.7 (62.3-70.6) | 66.8 (62.8-71.6) | 0.270 | 66.8 (62.7-71.0) | 66.8 (62.8-71.6) | 0.670 |
| Albumin/globulin | 1.10 (0.93-1.32) | 1.10 (0.93-1.32) | 1.12 (0.93-1.32) | 0.966 | 1.09 (0.92-1.29) | 1.12 (0.92-1.32) | 0.578 |
| T-Ch, mmol/L | 3.71 (3.09-4.39) | 3.73 (3.11-4.39) | 3.66 (3.05-4.43) | 0.863 | 3.75 (3.07-4.33) | 3.66 (3.05-4.45) | 0.943 |
| Triglyceride, mmol/L | 1.44 (1.13-1.85) | 1.44 (1.13-1.84) | 1.45 (1.15-1.89) | 0.294 | 1.43 (1.16-1.82) | 1.45 (1.16-1.91) | 0.426 |
| LDL, mmol/L | 2.14 (1.64-2.74) | 2.14 (1.64-2.72) | 2.15 (1.65-2.76) | 0.706 | 2.12 (1.64-2.70) | 2.15 (1.65-2.76) | 0.528 |
| HDL, mmol/L | 0.88 (0.73-1.03) | 0.88 (0.74-1.03) | 0.87 (0.70-1.02) | 0.125 | 0.89 (0.74-1.03) | 0.87 (0.70-1.02) | 0.228 |
| Creatinine, μmol/L | 80 (64-108) | 80 (64-109) | 78 (64-104) | 0.433 | 78 (63-106) | 78 (64-103) | 0.787 |
| BNU, mmol/L | 6.3 (4.7-9.1) | 6.3 (4.7-9.0) | 6.2 (4.5-9.4) | 0.305 | 6.3 (4.5-9.0) | 6.2 (4.5-9.4) | 0.940 |
| eGFR, mL/min/1.73m^2 | 80.5 (54.0-94.1) | 79.8 (53.0-94.0) | 81.3 (56.4-94.3) | 0.516 | 82.0 (54.8-95.3) | 81.0 (57.1-93.8) | 0.592 |
| Sodium, mmol/L | 137.7 (135.1-140.1) | 137.7 (135.1-140.1) | 137.7 (135.0-139.8) | 0.473 | 137.6 (134.9-139.9) | 137.7 (135.0-139.8) | 0.897 |
| Potassium, mmol/L | 4.09 (3.77-4.45) | 4.09 (3.78-4.46) | 4.07 (3.73-4.43) | 0.494 | 4.12 (3.77-4.47) | 4.07 (3.73-4.43) | 0.421 |
| APTT | 36.1 (32.7-40.0) | 36.1 (32.7-39.9) | 35.7 (32.4-40.6) | 0.650 | 36.1 (32.7-40.2) | 35.8 (32.4-40.7) | 0.965 |
| Prothrombin time, s | 13.3 (12.5-14.1) | 13.3 (12.5-14.1) | 13.2 (12.4-14.1) | 0.331 | 13.3 (12.4-14.1) | 13.2 (12.4-14.2) | 0.690 |
| Thrombin time, s | 17.6 (16.4-18.8) | 17.6 (16.4-18.8) | 17.6 (16.2-18.8) | 0.653 | 17.6 (16.2-18.8) | 17.7 (16.3-18.9) | 0.663 |
| International normalized ratio | 1.03 (0.97-1.11) | 1.03 (0.97-1.11) | 1.03 (0.96-1.11) | 0.909 | 1.03 (0.97-1.11) | 1.03 (0.96-1.11) | 0.585 |
| Fibrinogen, mg/L | 4.33 (3.52-5.27) | 4.34 (3.52-5.25) | 4.30 (3.50-5.29) | 0.688 | 4.39 (3.56-5.42) | 4.29 (3.46-5.28) | 0.230 |
| D-dimer, ug/mL FEU | 0.91 (0.51-1.80) | 0.90 (0.50-1.82) | 0.93 (0.55-1.75) | 0.622 | 0.87 (0.49-1.88) | 0.94 (0.55-1.72) | 0.430 |

Data were presented as median and interquartile range (Q1-Q3) or n(%). Keys: PSM, propensity score matching; CHD, coronary heart disease; CHF, chronic heart failure; T2DM, type 2 diabetes; CKD, chronic kidney disease; RR, respiratory rate; SBP, systolic blood pressure; DBP, diastolic blood pressure; hs-cTnI, high-sensitivity-cardiac troponin I; HbA1c, glycated hemoglobin/hemoglobin A1c; RBC, red blood cell; WBC, white blood cell; ALT, alanine aminotransferase; AST, aspartate aminotransferase; LDH, Lactate dehydrogenase; T-Ch, total cholesterol; LDL, low-density lipoprotein; HDL, high-density lipoprotein; BUN, blood urea nitrogen; eGFR, estimated glomerular filtration rate; APTT, activated partial thromboplastin time.

**Table S4. Long term medication of surviving COVID-19 patients in myocardial injury group and no injury group during one-year follow-up.**

|  | **All patients (n=2185)** | **Unmatched** | | | **PSM (1:1)** | | |
| --- | --- | --- | --- | --- | --- | --- | --- |
|  |  | **No injury group (n=1573)** | **Myocardial injury group (n=447)** | **p** | **No injury group (n=427)** | **Myocardial injury group (n=427)** | **p** |
| ACEI/ARB, n (%) | 445 (21.9) | 337 (21.4) | 108 (24.2) | 0.218 | 105 (24.6) | 99 (23.2) | 0.630 |
| Diuretic, n (%) | 426 (21.2) | 340 (21.6) | 86 (19.2) | 0.277 | 88 (20.6) | 82 (19.2) | 0.607 |
| CCB, n (%) | 615 (30.4) | 485 (30.8) | 130 (29.1) | 0.478 | 127 (29.7) | 127 (29.7) | >0.999 |
| β-blocker, n (%) | 396 (19.6) | 303 (19.3) | 93 (20.8) | 0.468 | 87 (20.4) | 89 (20.8) | 0.866 |
| Statins, n (%) | 482 (23.9) | 383 (24.3) | 99 (22.1) | 0.335 | 112 (26.2) | 93 (21.8) | 0.128 |
| Metformin, n (%) | 150 (7.4) | 120 (7.6) | 30 (6.7) | 0.514 | 37 (8.7) | 28 (6.6) | 0.245 |
| Nitrates, n (%) | 144 (7.1) | 115 (7.3) | 29 (6.5) | 0.551 | 27 (6.3) | 27 (6.3) | >0.999 |
| Anticoagulant drugs, n (%) | 72 (3.6) | 55 (3.5) | 17 (3.8) | 0.758 | 17 (4.0) | 17 (4.0) | >0.999 |
| Antiplatelet drugs, n (%) | 378 (18.7) | 295 (18.8) | 83 (18.6) | 0.929 | 83 (19.4) | 78 (18.3) | 0.662 |

Keys: PSM, propensity score matching; ACEI/ARB, angiotensin-converting enzyme inhibitors/angiotensin II receptor blockers; CCB, Calcium channel blockers.

**Symptom questionnaire for COVID-19 survivors at 12-month visit**

1. Have you experienced fatigue or muscle weakness now?

□ No (go Q2)	□ Yes (go Q1.1)

1.1 Is it newly onset post COVID-19?

□ No (go Q1.2)	□ Yes (go Q2)

1.2 Is it worse than the status prior to COVID-19?

□ No	□ Yes

2. Have you experienced sleep difficulty now?

□ No (go Q3)	□ Yes (go Q2.1)

2.1 Is it newly onset post COVID-19?

□ No (go Q2.2)	□ Yes (go Q3)

2.2 Is it worse than the status prior to COVID-19?

□ No	□ Yes

3. Have you experienced hair loss now?

□ No (go Q4)	□ Yes (go Q3.1)

3.1 Is it newly onset post COVID-19?

□ No (go Q3.2)	□ Yes (go Q4)

3.2 Is it worse than the status prior to COVID-19?

□ No	□ Yes

4. Have you experienced smell disorder now?

□ No (go Q5)	□ Yes (go Q4.1)

4.1 Is it newly onset post COVID-19?

□ No (go Q4.2)	□ Yes (go Q5)

4.2 Is it worse than the status prior to COVID-19?

□ No	□ Yes

5. Have you experienced palpitation now?

□ No (go Q6)	□ Yes (go Q5.1)

5.1 Is it newly onset post COVID-19?

□ No (go Q5.2)	□ Yes (go Q6)

5.2 Is it worse than the status prior to COVID-19?

□ No	□ Yes

6. Have you experienced joint pain now?

□ No (go Q7)	□ Yes (go Q6.1)

6.1 Is it newly onset post COVID-19?

□ No (go Q6.2)	□ Yes (go Q7)

6.2 Is it worse than the status prior to COVID-19?

□ No	□ Yes

7. Have you experienced decreased appetite now?

□ No (go Q8)	□ Yes (go Q7.1)

7.1 Is it newly onset post COVID-19?

□ No (go Q7.2)	□ Yes (go Q8)

7.2 Is it worse than the status prior to COVID-19?

□ No	□ Yes

8. Have you experienced taste disorder now?

□ No (go Q9)	□ Yes (go Q8.1)

8.1 Is it newly onset post COVID-19?

□ No (go Q8.2)	□ Yes (go Q9)

8.2 Is it worse than the status prior to COVID-19?

□ No	□ Yes

9. Have you experienced dizziness now?

□ No (go Q10)	□ Yes (go Q9.1)

9.1 Is it newly onset post COVID-19?

□ No (go Q9.2)	□ Yes (go Q10)

9.2 Is it worse than the status prior to COVID-19?

□ No	□ Yes

10. Have you experienced nausea or vomiting now?

□ No (go Q11)	□ Yes (go Q10.1)

10.1 Is it newly onset post COVID-19?

□ No (go Q10.2)	□ Yes (go Q11)

10.2 Is it worse than the status prior to COVID-19?

□ No	□ Yes

11. Have you experienced chest pain now?

□ No (go Q12)	□ Yes (go Q11.1)

11.1 Is it newly onset post COVID-19?

□ No (go Q11.2)	□ Yes (go Q12)

11.2 Is it worse than the status prior to COVID-19?

□ No	□ Yes

12. Have you experienced sore throat or difficult to swallow now?

□ No (go Q13)	□ Yes (go Q12.1)

12.1 Is it newly onset post COVID-19?

□ No (go Q12.2)	□ Yes (go Q13)

12.2 Is it worse than the status prior to COVID-19?

□ No	□ Yes

13. Have you experienced skin ras now?

□ No (go Q14)	□ Yes (go Q13.1)

13.1 Is it newly onset post COVID-19?

□ No (go Q13.2)	□ Yes (go Q14)

13.2 Is it worse than the status prior to COVID-19?

□ No	□ Yes

14. Have you experienced myalgia now?

□ No (go Q15)	□ Yes (go Q14.1)

14.1 Is it newly onset post COVID-19?

□ No (go Q14.2)	□ Yes (go Q15)

14.2 Is it worse than the status prior to COVID-19?

□ No	□ Yes

15. Have you experienced headache now?

□ No	□ Yes (go Q15.1)

15.1 Is it newly onset post COVID-19?

□ No (go Q15.2)	□ Yes

15.2 Is it worse than the status prior to COVID-19?

□ No	□ Yes

**Supplementary Table 3** Modified British Medical Research Council. (mMRC) scale

| mMRC grade | Evaluation criteria |
| --- | --- |
| 0 | Not troubled by breathlessness except with strenuous exercise. |
| 1 | Short of breath when hurrying on the level or walking up a slight hill. |
| 2 | Walks slower than people of the same age on the level because of breathlessness, or has to stop for breath when walking at own pace on the level. |
| 3 | Stops for breath after walking about 100 meters or after a few minutes on the level. |
| 4 | Too breathless to leave the house, or breathless when dressing or undressing. |

**Supplementary Table 4** EuroQol five-dimension five-level (EQ-5D-5L) questionnaire

| Dimensions | Levels |
| --- | --- |
| Mobility | (1) No problems with walking around  (2) Slight problems with walking around  (3) Moderate problems with walking around  (4) Severe problems with walking around  (5) Unable to walk around |
| Personal care | (1) No problems washing or dressing  (2) Slight problems washing or dressing  (3) Moderate problems washing or dressing  (4) Severe problems washing or dressing  (5) Unable to wash or dress |
| Usual activities (e.g., work, study, housework, family, or leisure activities) | (1) No problems with usual activities  (2) Slight problems with usual activities  (3) Moderate problems with usual activities  (4) Severe problems with usual activities  (5) Unable to do usual activities |
| Pain/discomfort | (1) No pain or discomfort  (2) Slight pain or discomfort  (3) Moderate pain or discomfort  (4) Severe pain or discomfort  (5) Extreme pain or discomfort |
| Anxiety/depression | (1) No anxious or depressed  (2) Slightly anxious or depressed  (3) Moderately anxious or depressed  (4) Severely anxious or depressed  (5) Extremely anxious or depressed |
